# Past, current, and potential future distributions of unique genetic diversity in a cold‐adapted mountain butterfly

**DOI:** 10.1002/ece3.6755

**Published:** 2020-09-30

**Authors:** Melissa Minter, Kanchon K. Dasmahapatra, Chris D. Thomas, Mike D. Morecroft, Athayde Tonhasca, Thomas Schmitt, Stefanos Siozios, Jane K. Hill

**Affiliations:** ^1^ Leverhulme Centre for Anthropocene Biodiversity Department of Biology University of York York UK; ^2^ Natural England York UK; ^3^ Scottish Natural Heritage Perth UK; ^4^ Senckenberg Deutsches Entomologisches Institut Müncheberg Germany; ^5^ Institute of Integrative Biology University of Liverpool Liverpool UK

**Keywords:** butterfly, climate change, genetic diversity, Last Glacial Maximum, mountain systems, Refugia

## Abstract

**Aim:**

Climatic changes throughout the Pleistocene have strongly modified species distributions. We examine how these range shifts have affected the genetic diversity of a montane butterfly species and whether the genetic diversity in the extant populations is threatened by future climate change.

**Location:**

Europe.

**Taxon:**

*Erebia epiphron* Lepidoptera: Nymphalidae.

**Methods:**

We analyzed mtDNA to map current genetic diversity and differentiation of *E. epiphron* across Europe to identify population refugia and postglacial range shifts. We used species distribution modeling (SDM) to hindcast distributions over the last 21,000 years to identify source locations of extant populations and to project distributions into the future (2070) to predict potential losses in genetic diversity.

**Results:**

We found substantial genetic diversity unique to specific regions within Europe (total number of haplotypes = 31, number of unique haplotypes = 27, *H*_d_ = 0.9). Genetic data and SDM hindcasting suggest long‐term separation and survival of discrete populations. Particularly, high rates of unique diversity in postglacially colonized sites in England (*H*_d_ = 0.64) suggest this population was colonized from a now extinct cryptic refugium. Under future climate change, SDMs predict loss of climate suitability for *E. epiphron*, particularly at lower elevations (<1,000 meters above sea level) equating to 1 to 12 unique haplotypes being at risk under climate scenarios projecting 1°C and 2–3°C increases respectfully in global temperature by 2070.

**Main conclusions:**

Our results suggest that historical range expansion and retraction processes by a cold‐adapted mountain species caused diversification between populations, resulting in unique genetic diversity which may be at risk if distributions of cold‐adapted species shrink in future. Assisted colonizations of individuals from at‐risk populations into climatically suitable unoccupied habitat might help conserve unique genetic diversity, and translocations into remaining populations might increase their genetic diversity and hence their ability to adapt to future climate change.

## Graphical abstract

The genetic diversification of cold‐adapted mountain species, such as *E. epiphron*, has been shaped by Pleistocene glaciations, resulting in unique genetic diversity in isolated populations. The unique genetic diversity in mountain and cold‐adapted species is at under future climate warming, and we predict *E. epiphron* will lose 38%–64% of its range in the future, resulting in the loss of genetic diversity, reducing its ability to adapt.

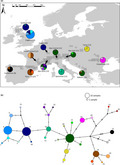

## 1 INTRODUCTION

Projecting the future geographic distribution of genetic variation within species’ ranges and the potential loss of genetic variation from anthropogenic climate change requires understanding of the past, present, and future distributions of species (Wroblewska & Mirski, 2018). Geographic variation in the distribution of genes across a species’ range results from a combination of historical and current conditions, which influence patterns of genetic differentiation among populations that are, or have been, geographically isolated, and from colonization bottlenecks during range shifts (Hewitt, 2004). These range shifts and their genetic consequences have primarily been driven by the fundamental niche of a species, or their “climate‐envelope,” and species’ ranges shift to track environmental changes, altering the location of populations and their genetic structure (Hewitt, 2004; McCallum, Guerin, Breed, & Lowe, 2014; Thomas, 2010). The Earth has gone through many climate fluctuations, including glaciations in the Pleistocene and human‐induced climate change in the current Anthropocene (Hewitt, 2004; Santer et al., 2019). Future anthropogenic climate warming may further impact species through distribution changes, genetic erosion, and extinctions (Botkin et al., 2007). Cold‐adapted/mountain species may be especially vulnerable to future climate changes as they are already restricted to mountain ecosystems where suitable climate space is limited, and loss of genetic diversity within these range‐restricted cold‐adapted species may reduce their ability to adapt to future changes (Elsen & Tingley, 2015). Understanding how past climatic changes have impacted current genetic structure may allow us to make predictions for the likely extent of genetic loss under future climate change and thereby prioritize at‐risk populations for conservation management (McCallum et al., 2014; Wroblewska & Mirski, 2018).

During the last ice age, ice sheets were at their greatest extension 20,000–21,000 years ago, during the Last Glacial Maximum (LGM) (Crowley & North, 1991; Ray & Adams, 2001). During the LGM, species were thought to persist where climatic conditions were buffered, at lower elevations or in more southerly regions (Dapporto et al., 2019; Morelli et al., 2016); however, some studies have shown evidence of species surviving in northern isolated refugia (Provan & Bennett, 2008; Schmitt & Varga, 2012; Stewart & Lister, 2001). Cold‐adapted species which currently occur in mountain ecosystems were probably more widespread during the LGM and only became isolated in their current interglacial populations after climate‐induced range retraction, although some cold‐adapted species were already restricted to isolated glacial refugia during the LGM (Schmitt, 2009; Schmitt, Hewitt, & Muller, 2006). The consequences of past distribution changes will be reflected in current genetic diversity, because contractions and expansions from long‐term refugia leave a genetic signature of high diversity in refugia compared to lower diversity in recently colonized populations (Hewitt, 2000; Keppel et al., 2012; Morelli et al., 2016). Thus, understanding historical interactions of cold‐adapted species with climate can help us understand current genetic structure and diversity of populations.

Lepidoptera are poikilothermic and therefore sensitive to changes in climate, and those species which are cold‐adapted are particularly vulnerable to warmer conditions (Deutsch et al., 2008; Elsen & Tingley, 2015). Some cold‐adapted Lepidoptera are experiencing extinctions at their low latitude/elevation margins as the climate deteriorates for these species (Franco et al., 2006; Wilson, Gutierrez, Gutierrez, & Monserrat, 2007). The Mountain Ringlet *Erebia epiphron* is a butterfly found in the mountains of continental Europe and Britain, and its distribution has retracted 130–150 m uphill in Britain over the past five decades due to climate warming (Franco et al., 2006). Therefore, *E. epiphron* represents a good model organism to understand how past climate‐induced changes have impacted current genetic structures of populations, and whether genetic diversity may be lost with further climate‐induced local extinctions.

Species distribution modeling (SDMs) are commonly used to project future distributions of species under climate change scenarios (Guo et al., 2017; Urban, 2015) and to develop climate adaptation conservation strategies. These modeling approaches have also been used with paleoclimate data to hindcast past distributions and to understand how they shape current population structures (Smith, Gregory, Anderson, & Thomas, 2013). Phylogeography with genetic techniques can be used to identify divergence between populations and to infer historical distribution patterns and colonization routes (Luquet et al., 2019). Previous studies have shown how a combination of species distribution modeling and phylogeography can provide better understanding of past, present, and future distributions of species and predict the potential loss of genetic diversity resulting from climatic warming (Schmitt, Habel, Rodder, & Louy, 2014; Wroblewska & Mirski, 2018; Yannic et al., 2014).

In this study, we use mtDNA sequencing to map the current distribution of genetic diversity of the cold‐adapted butterfly, *E. epiphron*, and also use species distribution modeling to project current, past, and future distributions of the species. We use this genetic and modeling information to determine the distribution of *E. epiphron* in continental Europe during the Last Glacial Maximum, the locations of glacial refugia, and patterns of subsequent postglacial expansion into northerly latitudes in Britain. We identify populations with unique genetic diversity and examine potential loss of genetic diversity under future climate change scenarios in order to prioritize populations for protection.

## 2 METHODS

### 2.1 Genetic analyses to map current haplotype diversity

We sampled 146 adults of *E. epiphron* from 13 mountain regions across continental Europe and Britain. European populations (76 adults) were sampled between July and August 2002–2014, populations in England and Scotland (74 adults) were sampled in June–July 2016–2019, and adults preserved in 100% ethanol at −20°C. All relevant fieldwork permissions were obtained. DNA was extracted from 111 individuals with Omega Bio‐tek E.Z.N.A.® DNA Isolation Kit following the manufacturer's protocol. For each individual, the head and antennae were removed and placed in 1.5 ml tubes with CLT buffer and proteinase K and homogenized with pellet pestles. A 658‐bp fragment of the mitochondrial cytochrome oxidase‐I (COI) gene was amplified using the primers LepF (5’‐ATTCAACCAATCATAAAGATATTGG‐3’) and LepR (5’‐TAAACTTCTGGATGTCCAAAAAATCA‐3’) (Hajibabaei, Janzen, Burns, Hallwachs, & Hebert, 2006). PCR amplification of individual DNA samples was carried out in 20 µl reactions which included 1.8 µl of template DNA, 1x PCR reaction buffer (Promega), 1.5 mM MgCl_2_, 0.2 mM of dNTPs, and 1U of *Taq* DNA polymerase (Promega GoTaq®). PCR conditions used the following profile: 94°C for 2 min (one cycle), 2 min at 94°C, 58°C for 45 s, and 72°C for 1 min (35 cycles), followed by a final extension step of 75°C for 5 min. PCR products were purified and Sanger sequenced with forward and reverse primers using © Eurofins Scientific PlateSeq service and LightRun Tube service. Chromatograms were checked visually using SeqTrace (Stucky, 2012). Additional COI sequences were obtained from a panel of 39 samples collected in England in June 2016 as a part of a whole‐genome resequencing project (NERC highlight project NE/N015797/1). Briefly, the complete mitochondrial genome was assembled for each individual sample using the MitoZ toolkit (Meng, Li, Yang, & Liu, 2019) and annotated using the mitos2 web server (Bernt et al., 2013). Low coverage regions (<10) were masked to avoid introducing low‐quality SNPs and the COI region was extracted for further analyses.

These 150 sequences along with 65 existing COI sequences from GenBank were combined to create a data set of 215 COI sequences from 13 mountain regions across the species’ European range (for sample information see Appendix S1 and map of mountain regions see Appendix S2). These sequences were aligned with ClustalX implemented in MEGA‐X (Kumar, Stecher, Li, Knyaz, & Tamura, 2018) and the alignment checked by eye and cropped to the same length (649 bp). Haplotypes were identified and genetic diversity measures were determined using DnaSP 6 (Rozas et al., 2017). Genetic diversity measures included number of haplotypes (*H*_n_), number of unique haplotypes (*H*_u_), haplotype diversity (*H*_d,_ the probability that two randomly sampled alleles are different), and nucleotide diversity (*π*, the average number of nucleotide differences per site between sequences (Nei, 1987). A TCS network (Templeton, Crandall, & Sing, 1992) of all haplotypes was constructed using PopArt (Leigh & Bryant, 2015). A CO1 phylogenetic tree was constructed in BEAST (Suchard et al., 2018) of the Erebia genus, outgroups, and the *E. epiphron* populations. The same methods and CO1 sequences were used from (Peña, Witthauer, Klečková, Fric, & Wahlberg, 2015) using a log‐normal relaxed molecular clock, with a birth–death incomplete speciation model for the randomly generated tree prior, and then an uncorrelated log‐normal relaxed molecular clock and all the programs other default settings to model the rate of evolution. The age between Erebia and its sister taxa was set at 37.4 ± 2 Myr, (Peña et al., 2015) to estimate age in divergence between *E. epiphron* subpopulations.

### 2.2 Using species distribution modeling (SDMs) to map current distribution of *E. epiphron*

Current distribution data for *E. epiphron* (50 × 50‐km grid resolution) were obtained from the Distribution Atlas of European butterflies (http://www.ufz.de/european‐butterflies/index.php?en=42605). Current (1970–2000) climate data were downloaded from WorldClim (http://www.worldclim.org/) at a resolution of 2.5 arc minutes (~4.5‐km grid cell resolution). Climate variables for inclusion in SDMs were selected to reflect climate limitations and extremes of cold‐adapted species, which are likely to be most limited by climatic conditions during the coldest and hottest times of the year. We therefore included climate data on annual mean temperature and mean precipitation of the coldest quarter (December to February) and warmest quarter (June to August) of the year (Smith et al., 2013). Spatial autocorrelation was tested using Moran's I in R. The butterfly distribution data were at 50‐km grid resolution, but the species is likely to be restricted by local climate conditions in each grid square (Smith et al., 2013). Thus, we included in models only the coldest/warmest and wettest/driest cells (4.5‐km resolution) within each 50‐km grid, resulting in a total of eight climatic variables being incorporated into our SDMs (see Appendix S3). 50 × 50‐km grid cell resolution data are appropriate for our model building to address biogeographic questions at regional scales, because we are interested in changes in the distribution of the study species over long periods of time (i.e., millennia), rather than shorter‐term changes at individual sites. This 50‐km spatial resolution also ensures that the pseudo‐absences (i.e., locations where *E. epiphron* is assumed to be absent) are more accurate representations of true absences, because these grids have been visited by butterfly recorders but *E. epiphron* was not recorded as present. In addition, 50‐km data for presences cover the entire global distribution of *E. epiphron at* this spatial resolution. Butterfly distributions were modeled using an ensemble approach (R package BIOMOD2; (Thuiller, Lafourcade, Engler, & Araujo, 2009), combining outputs from the models; generalized linear models (GLM), multiple adaptive regression splines (MARS), maximum entropy (MAXENT.Phillips), generalized additive model (GAM), boosted regression trees (GBM), classification tree analysis (CTA), artificial neural network (ANN), surface range envelope (SRE), flexible discriminant analysis (FDA), and random forest (RF). We used the mean receiver operating characteristic (ROC) value to evaluate each model, with a threshold of ROC > 0.85 for inclusion of models within the ensemble model. We restricted pseudo‐absences to locations within a buffer of 250 km around presence data points to avoid placing absences in mountain systems with potentially suitable climate space that are not currently occupied by the species (e.g., Scandinavia) (Akcakaya & Atwood, 1997; Hirzel, Helfer, & Metral, 2001). Models were generated using 70% training data and 30% testing data (Franklin, 2010; Huberty, 1994).

### 2.3 Hindcasting past distributions and identifying glacial refugia

We incorporated paleoclimate data into our ensemble SDM for the eight climate variables representing the coldest/hottest and driest/wettest locations within each 50‐km grid square. Data for climate projections over the last 21,000 years were downloaded from PaleoView (2.5 × 2.5° (latitude/longitude) grid) (Fordham et al., 2017), and downscaled to match the resolution of the current climate data (2.5 arc minutes), using established methods (Mitasova & Mitas, 1993; Platts, Omeny, & Marchant, 2015; Ramirez‐Villegas & Jarvis, 2010). We projected climate suitability for *E. epiphron* every 1,000 years from the LGM to 1,000 years before present, generating 21 outputs, which were each clipped using Eurasian ice sheet data (Hughes, Gyllencreutz, Lohne, Mangerud, & Svendsen, 2016). Long‐term climate suitability of 50‐km grid squares was calculated by overlaying the 22 output maps and summing the climate suitability probability values of each grid, and then designating the top 30% of grids with highest probability values as areas of highest long‐term climate stability for the study species (Chan, Brown, & Yoder, 2011).

### 2.4 Projecting future distributions and loss of genetic diversity

**FIGURE 1:**
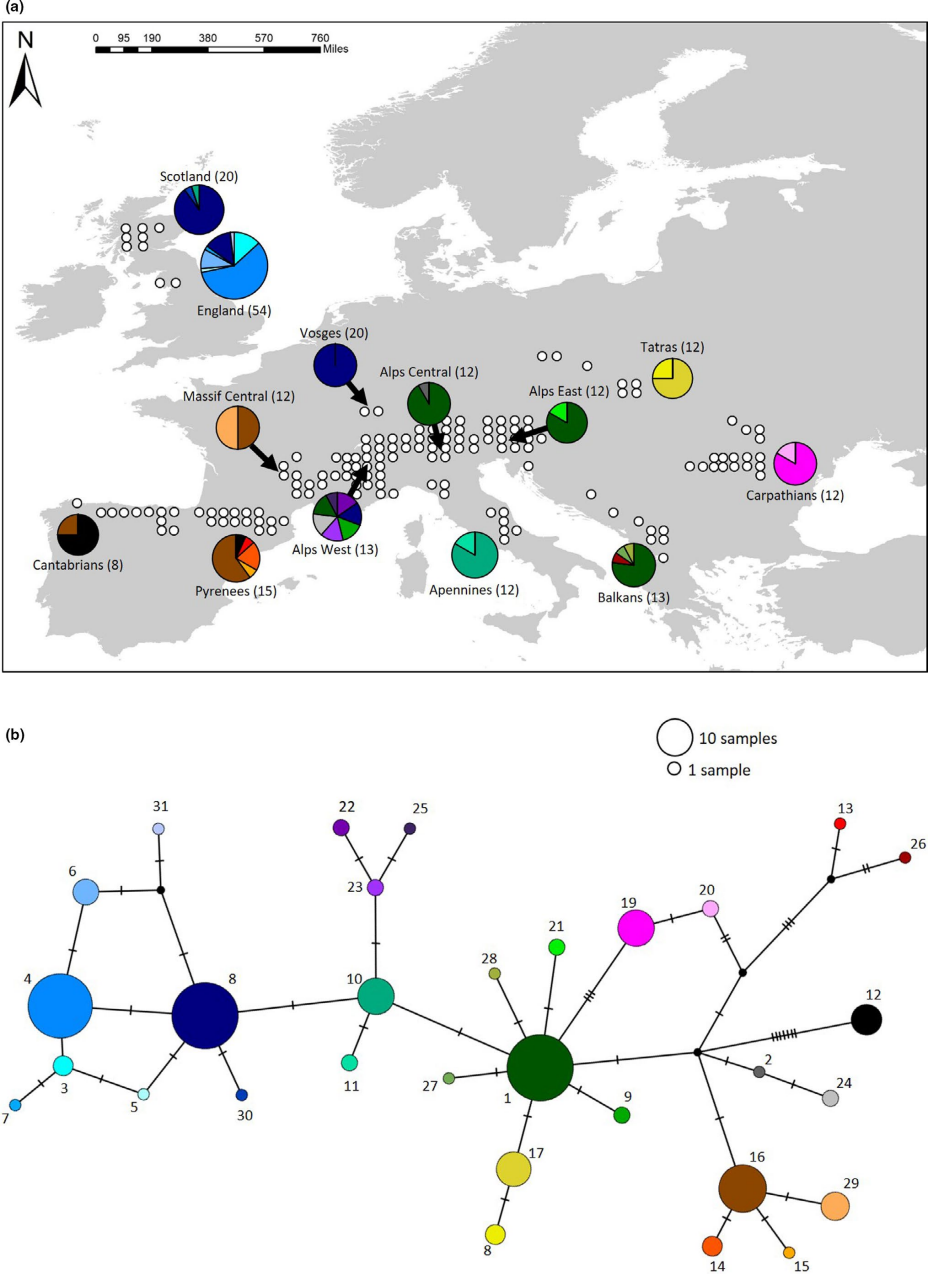
Current distribution of genetic diversity of *E. epiphron* and historical divergence. (a) Frequency pie charts of haplotypes across the species’ European range, including the current observed distribution of *E. epiphron* (white circles; 50‐km resolution) in 13 mountain regions, with number of samples (individuals) in brackets. (b) TCS network of all 31 identified haplotypes. Size of circle represents number of individuals containing that haplotype and tick marks represent a nucleotide substitution

Future climate projections for 2070 were obtained from IPCC 5th Assessment Report (Complete Coupled System Model, CCSM4 global climate models) from WorldClim (http://www.worldclim.org/; 2.5 arc minutes resolution) for high (RCP 8.5, ~2–3°C warming) and low (RCP 2.6, ~1°C warming) future climate scenarios. Unique haplotypes were assumed to be at risk if all 50‐km grid squares in one of the 13 mountain regions were predicted to become climatically unsuitable in the future (based on binary presence or absence threshold probability values from the ensemble SDM output). We set the threshold value as the probability value associated with the low elevation climatic range edge *E. epiphron* in its current range (low elevation range boundary in England; threshold probability = 0.49). Using this threshold, model probabilities were converted into the presence/absence to show grid squares with no change over time (i.e., population persistence), grids predicted to become climatically unsuitable (i.e., extinction), and grids predicted to become climatically suitable (i.e., colonization). Haplotype risk (*H_r_*) was calculated as the number of unique haplotypes at risk in each of the 13 mountain regions (Figure 1a) due to projected loss of all climatically suitable areas within a region in the future.

## 3 RESULTS

### 3.1 Current haplotype diversity across 13 mountain regions in Europe

From our 215 mtDNA samples, we identified 31 mtDNA haplotypes across Europe, including 27 haplotypes unique to a specific mountain region (Figure 1a, Table 1). The high frequency of unique haplotypes across Europe suggests low levels of allele sharing. There was also high genetic differentiation between populations (AMOVA, ϕ = 0.76, *p* < .001) and the divergence between some of these populations is dated before the Last Glacial Maximum (phylogenetic tree: see Appendix S5). The mountain regions containing the highest haplotype diversity include the Pyrenees (*H*_d_ = 0.63) the western Alps (*H*_d_ = 0.91) and England (*H*_d_ = 0.64) (Table 1). The mountain regions containing only unique haplotypes include the Carpathians (*H*_u_ = 2) and the Tatras (*H*_u_ = 2). Populations in England (*H*_u_ = 6) and the western Alps (*H*_u_ = 6) not only had the highest number of unique haplotypes but also contained some shared haplotypes with other regions (Figure 1a). There are six unique haplotypes in England which diverged from haplotype 8 (Figure 1b), which is present in England, Scotland, Vosges, and the western Alps. None of the 6 unique haplotypes in England were found anywhere else, although the presence of the shared haplotype 8 suggests historical allele sharing with the western Alps. Scotland, in addition to the shared haplotype 8, contains one unique haplotype (haplotype 30), which has diverged from haplotype 8 by 1 substitution and shares haplotype 10 with the Apennines (Figure 1). Despite evidence that regions are differentiated, shared haplotypes also provide evidence of historical gene flow across Pyrenees and Cantabrians, and between the Alps and Balkans (Figure 1). The Massif Central population shares one haplotype (haplotype 16) with the Pyrenees and Cantabrian Mountains, and has one unique haplotype (haplotype 29) which diverged from haplotype 16 by one substitution (Figure 1b).

**TABLE 1:** Current genetic diversity, and projected loss of climate suitability and haplotype loss in the future (2070)

Region	Current genetic diversity				% Range change (low)	% Range change (high)	Haplotypes at risk	
	*H*_n_	*H*_u_	*H*_d_	*π*			*H*_r_ (low)	*H*_r_ (high)
All	31	27	0.89	0.0055	−38.6	−64.3	1	12
Vosges	1	0	0	0	−100	−100		
Scotland	3	1	0.194	0.0003	−37.5	−25		
Pyrenees	5	3	0.629	0.004	−20	−73.3		
Massif Central	2	1	0.545	0.0008	No change	−50		
England	7	6	0.638	0.0015	−50	−100		6
Carpathians	2	2	0.303	0.0005	−70.6	−100		2
Tatras	2	2	0.409	0.0006	−25	−75		
Cantabrians	2	0	0.429	0.0059	−63.6	−81.8		
Balkans West	4	3	0.423	0.0024	−75	−100		3
Apennines	2	1	0.303	0.0005	−100	−100	1	1
Alps West	7	5	0.912	0.0043	−14.3 (all Alps)	−41.3 (all Alps)		
Alps East	2	1	0.303	0.0005				
Alps Central	2	1	0.182	0.0006				

Note

*H*_n_ = number of haplotypes; *H*_u_ = number of unique haplotypes; π = Nei nucleotide diversity (Pi); % range change = % change in range size (number of occupied 50‐km grid squares) in the future compared with current distribution, and *H*_r_ = number of unique haplotypes at risk in the future, under RCP 2.6 (low) and 8.5 (high) climate scenarios.

### 3.2 Modeling the current distribution of *E. epiphron*

**FIGURE 2:**
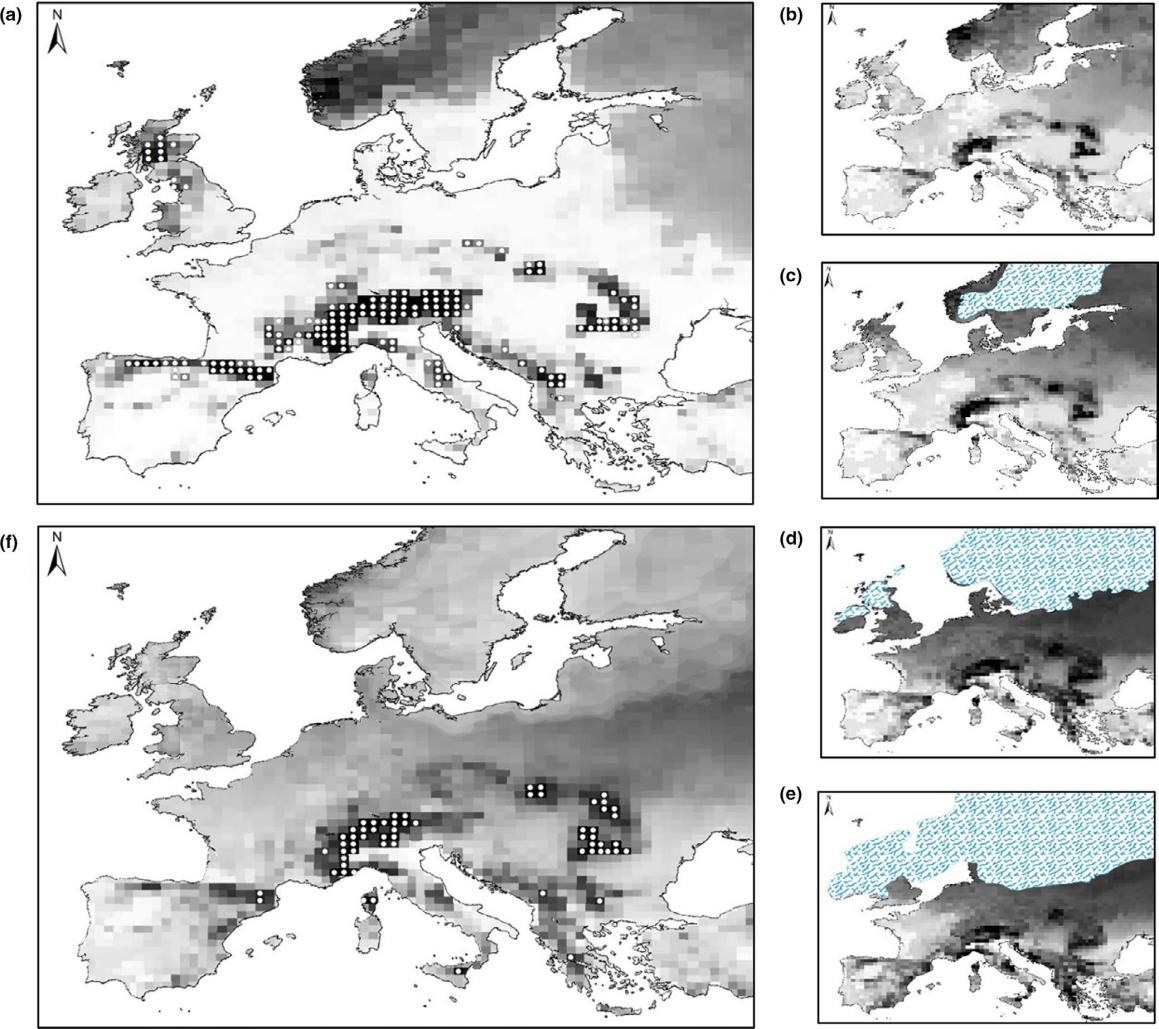
Current and past projected distributions of *E. epiphron*, (a) current probability of climate suitability and current distribution records (white circles). Past climate suitability (b) 6,000 years ago, (c) 11,000 years ago, (d) 16,000 years ago, and (e) 21,000 years ago (i.e., LGM; blue shading shows the extent of the ice sheet (from Hughes et al., 2016). Probability values of occurrence for b‐e scaled from 0 (unsuitable, white) to 1 (suitable, black). Panel f shows climate stability over time since the LGM produced by summing 22 outputs from SDMs for the last 21,000 years, plus the output for the present (summed probability values scaled from 0.73 (white) to 20 (black), with the top 30% of grids shown as white circles). See Appendix S4 for all output maps

Our ensemble SDM was a good fit to the current distribution of *E. epiphron* (95.4% of the presences predicted correctly, 76.3% of pseudo‐absences predicted correctly (based on the total presence data), ROC = 0.9) (Figure 2a). Areas predicted to be climatically suitable but currently uninhabited by *E. epiphron* include Wales, Scandinavian mountains, and eastern Balkans, the latter of which is currently occupied by *Erebia orientalis*. The model rated the minimum temperature of the warmest quarter of the year (June–August) as the most important variable for predicting climate suitability for the species (average importance of this variable across models = 0.73; importance rated from 0–1), probably because this is an important variable in identifying high elevation areas within a 50‐km grid square.

### 3.3 Hindcasting past distributions of *E. epiphron* and identifying areas of long‐term survival

Climate suitability in the LGM (21,000 years before present) showed overlap of climatically suitable areas (at 50‐km grid resolution) with many locations currently occupied by *E. epiphron*, as well as some southerly locations (Figure 2e). This overlap was confirmed when all 21 SDM outputs for each 1,000‐year time period up to the present day were combined to show long‐term climatic stability since the LGM (Figure 2f). These climate stability maps provided evidence that the locations of glacial refugia were in areas of high topographic variation within the species’ current distribution in continental Europe.

### 3.4 Projecting future distributions and loss of genetic diversity

**FIGURE 3:**
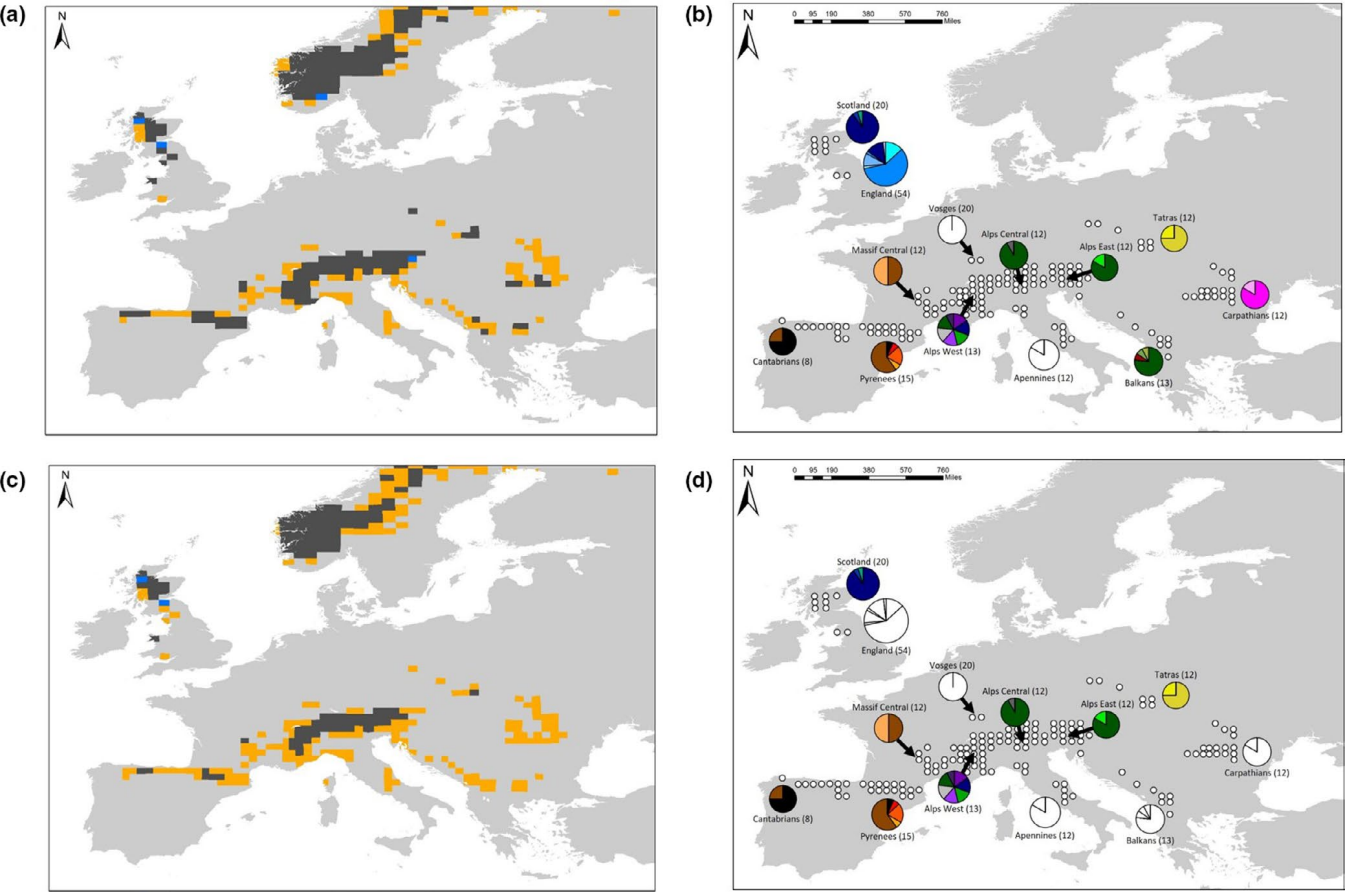
Projecting future climate suitability for *E. epiphron* in 2070 under two RCP climate change scenarios and associated projected loss of genetic diversity. (a) Low RCP 2.6 climate scenario (~1°C increase by 2070) and (c) high RCP 8.5 scenario (~2–3°C increase by 2070) showing grids projected to remain climatically suitable (black), become unsuitable (orange), and become suitable (blue). (b) Low RCP 2.6 scenario haplotype map with predicted lost haplotypes colored in white (2 regions lost, 1 unique haplotype lost), and D) high RCP 8.5 haplotype map with predicted lost haplotypes colored in white (5 regions lost, 12 unique haplotypes lost)

As expected for a cold‐adapted species, SDM outputs from both high and low future climate change scenarios project that many extant *E. epiphron* areas will have reduced climate suitability in the future (38%–64% loss of 7,000‐km^2^ occupied grids across Europe) (Figure 3, Table 1). The loss of climate suitability is most severe in lower elevation sites, as shown by significant linear regressions between change in probability over time and average elevation of the 50‐km grid square (low scenario: *p* < 0.001, *R* ^2^: 0.27, *F*_150 =_ 56.51; high scenario: *p* < 0.001, *R* ^2^: 0.13, *F*_150_ = 22.86). The mountain regions predicted to experience the greatest reduction in range size are the Vosges (100% loss of grid squares under both scenarios) and Apennines (100% loss of grid squares under both scenarios), followed by the Balkans (75%–100% loss), Carpathians (70%–100% loss), England (50%–100% lost), and Cantabrians (63%–81% loss) (Figure 3, Table 1). These range retractions result in the potential loss of 1 haplotype under the low climate change scenario (RCP 2.6), and the total loss of 12 unique haplotypes under the high climate change scenario (Figure 3, Table 1). Many of the haplotypes predicted to be lost are a single substitution from their nearest haplotype, however the haplotypes in the Carpathians are more genetically distinct (Figure 1b). By contrast, range sizes in the Alps and Scotland are projected to remain relatively stable, assuming the species colonizes sites at higher elevations that are predicted to become climatically suitable in these regions. Under both scenarios, areas north of Scotland and England become suitable in the future. Although *E. epiphron* does not currently occur in Scandinavia, our models predict that this area will remain stable in climate suitability in the future.

## 4 DISCUSSION

By using species distribution modeling and mtDNA analyses, we explore the past, present, and potential future distributions of genetic diversity in the cold‐adapted species *E. epiphron*. We identify high levels of genetic differentiation across Europe and found evidence of long‐term climate suitability in many of these regions since the LGM, which suggests these climatically stable regions were refugial areas of long‐term survival by our study species over the last 21,000 years and potentially longer‐term areas of persistence over previous glacial–interglacial cycles. Our study focuses on a single mountain species but our findings are likely to be widely applicable to other mountain species where populations contain unique genetic diversity as a consequence of past climate fluctuations, and which may be at risk under future climate warming. These areas of long‐term survival are within topographically heterogeneous landscapes, allowing populations to shift to the foothills during glacial periods. Our analyses also revealed that populations in the Massif Central, Vosges, and Britain are presumed postglacial colonizations (Figure 1, Figure 2f) due to low climate suitability over time, shared haplotypes, and the fact that Britain was under an ice sheet during the LGM. Britain was apparently colonized via two different routes, with the Scottish populations likely originating from populations in Vosges/Alps mountain regions due to the high prevalence of shared haplotype 8. By contrast, the English population has high levels of unique genetic diversity, and no evidence that any of the six unique haplotypes are shared with other extant populations (although there is one shared haplotype present), suggesting the English population has separated from the western Alps before the Last Glacial Maximum (given the large number of nucleotide substitutions; Figure 1b), and colonized Britain via a different route, from a cryptic refugium in an area where the study species survived during the glacial period but where it no longer exists. Under future climate change scenarios, we predict 38%–64% loss of range size, which equate to 1 unique haplotype to 12 unique haplotypes being at risk of loss under climate scenarios projecting 1°C and 2–3°C increases respectfully.

### 4.1 Limitations

This study has potential limitations, which are inherent in species distribution modeling, especially when projecting into different climates (Buisson, Thuiller, Casajus, Lek, & Grenouillet, 2010). We did not have suitable data to include sampling effort formally into our models, and so, the areas outside of the current *E. epiphron* distribution are considered “pseudo‐absences” rather than “true” absences. However, other butterfly species have been recorded in these squares (Lepidopterists have visited these squares) without recording *E. epiphron* as present, and hence, the proportion of false absences in the data is likely to be very low at the spatial (50 km across the whole of Europe) and temporal (accumulation of Lepidoptera records over 3 decades) scales considered here. We consider that our modeling approach robustly describes the bioclimatic conditions occupied by *E. epiphron* at a continental scale (the species’ global distribution). Future work could use sampling effort to account for imperfect species detection, with standardized sampling and occupancy modeling providing additional insight into (especially) within‐region distributions and dynamics.

For future projections, the loss of populations and consequently genetic diversity was based on a probability threshold to define butterfly presence or absence. This threshold was based on the probability value for English populations, given that this region represented the lowest elevational range edge for the study species. However, currently realized and fundamental niche characteristics may differ among regions (i.e., thresholds may differ), and hence, caution should be taken with our predictions. The difference between using two different thresholds (either the lowest elevation versus a threshold calculated by the Biomod2 program), affects whether or not the entire English and Apennines regions are lost, and hence, there is some uncertainty about the level of genetic diversity at risk. Nonetheless, the relatively low probability of future persistence in both of these regions suggests that these populations are at the climatic range limit for the species and therefore at risk. While regional adaptations may differ, we have no evidence that haplotypes are individually adaptive to climate variables, and hence, we use them as markers of colonization rather than as adaptive traits. For the same reason, we did not model the specific niches of individual haplotypes when considering the potential future loss of genetic variation (Breiner, Nobis, Bergamini & Guisan et al., 2018). Future work could use next‐generation sequencing to further test our hypotheses and to model‐specific genetic–climatic relationships in the future (see Bay et al., 2018).

Our analyses suggest that entire mountain regions of the butterfly's distribution could be lost under future climatic change, but it is possible that isolated populations could survive in particular microhabitats, at least temporarily. However, these localized populations may not contain all of the genetic variation currently present in the wider region, and overtime, these refugial populations may gradually lose genetic variation and viability (e.g., through inbreeding), and so, they may not persist in the longer term due to their isolation (metapopulation failure). A variety of processes may lead to the loss of genetic diversity following isolation, and there can sometimes be a delay in genetic loss following population decline (Kadlec, Vrba, Kepka, Schmitt, & Konvicka, 2010). For example, the sister species of *E. epiphron*, *Erebia orientalis*, is very localized and currently occurs only in the Eastern Balkans and is genetically homogeneous, potentially putting it at risk of inbreeding depression (Hinojosa, Monasterio, Escobes, Dinca, & Vila, 2019). Therefore, our model projections should be seen as representing much longer‐term regional‐scale expectations, rather than short‐term predictions at the local population or microhabitat scale. We believe that our conclusions about the long‐term (LGM to present) continental‐scale dynamics of *E. epiphron* are robust and that this knowledge of the past helps frame future risks and provides information for conservation management.

### 4.2 Long‐term survival resulting in unique genetic diversity in cold‐adapted species

SDM outputs provide evidence that our exemplar cold‐adapted study species occurred in disjunct regions throughout the period from the LGM to the present day, based on the distribution of suitable climate; the genetic data confirm likely separation not only since the LGM, but most probably over much longer periods and successive glacial–interglacial cycles. For mountain species, limited gene flow between the disjunctive parts of their range during glacial and interglacial periods results in divergence and unique haplotypes, unlike lowland European species which colonized northwards from their glacial refugia, and where large parts of the current geographic ranges often share haplotypes (Hewitt, 2004). Only limited areas of postglacial expansions and retractions are evident in *E. epiphron*, and the British populations would be susceptible to extinction if the climate was to return to LGM conditions at some time in the future. Similarly, our SDM outputs suggest that additional populations of *E. epiphron* could have existed further south in southern Europe at the LGM (Figure 2e) but as they no longer exist a northwards translocation of the range might have taken place under interglacial conditions. If cold‐adapted species such as *E. epiphron* were more widespread during glacial periods, then the current divergence could be associated with subsequent losses of genetic diversity (e.g., due to selection, or random drift during population bottlenecks), or a failure of our analyses to detect localized or rare haplotype variation. However, this alternative hypothesis seems unlikely because our estimates of times of genetic divergence (phylogenetic tree: see Appendix S5) imply that most splits occurred before the LGM. However, other divergence dates between *E. epiphron* and *E. orientalis* have been reported (e.g., 1.53 (±0.65) Mya (Hinojosa et al., 2019)). However, they still reported strong mtDNA divergence and long‐term separation (Hinojosa et al., 2019), and therefore, different assumptions of divergence dates do not impact the interpretation of our results. Hence we conclude that populations of *E. epiphron* survived as allopatric populations in mainland Europe during the LGM, with postglacial colonizations from these regions into the Massif Central, Vosges, Scotland, and England.

High genetic differentiation is observed among populations of other mountain *Erebia* species, supporting the hypothesis that they also survived as allopatric populations during the LGM (Haubrich & Schmitt, 2007; Louy, Habel, Abadjiev, et al., 2014; Louy, Habel, Ulrich, & Schmitt, 2014; Martin, Gilles, Lortscher, & Descimon, 2002; Schmitt et al., 2014; Schmitt, Louy, Zimmermann, & Habel, 2016; Schmitt & Seitz, 2001). LGM separation of populations has also been identified in mountain plants and other invertebrates (Bettin, Cornejo, Edwards, & Holderegger, 2007; Huck, Budel, & Schmitt, 2012; Margraf, Verdon, Rahier, & Naisbit, 2007; Pauls, Lumbsch, & Haase, 2006). The numbers of glacial–interglacial cycles over which populations have remained disjunct remain unclear, but some studies have indicated divergence dates covering several glacial–interglacial cycles or even predating the Pleistocene (Hewitt, 2000). The reality is likely to be more complex with areas of persistent separation, but with occasional links between them (i.e., rare gene flow or brief periods of connection), as indicated by the distributions and relatedness of haplotypes in Figure 1.

### 4.3 Unique haplotypes in populations derived from northern cryptic refugia

Following the LGM, the ice retreated in northern Europe and many species colonized northwards, for example, via the land bridge between continental Europe and Britain, which was present until sea level rise ~ 7,000 years before present (Sturt, Garrow, & Bradley, 2013). The locations of southerly glacial refugia, which are thought to be the main sources of colonizations, have been debated extensively, with proposed glacial refugia in the Iberian Peninsula, Italy, and the Balkans (Hewitt, 2000), and this has recently been reinforced in European butterflies (Dapporto et al., 2019). However, there is also evidence for more northern cryptic refugia based on fossil, pollen, and genetic evidence (Birks & Willis, 2008; Provan & Bennett, 2008; Stewart & Lister, 2001), where species apparently persisted at higher latitudes in sheltered locations with suitable microclimates (Stewart, Lister, Barnes, & Dalen, 2010). However, most cryptic refugia described to date have been for relatively warm‐adapted species. Here, we present evidence for the existence of northern cryptic population(s) for cold‐adapted species during the LGM, based on high unique genetic diversity of the present‐day *E. epiphron* populations in England, an area that was beneath an ice sheet at the LGM (Hughes et al., 2016). The high genetic uniqueness of populations in England, together with a single shared haplotype with Scotland/Vosges/Alps (haplotype 8; Figure 1b), is consistent with northern colonizations from the Alps, but distinct separate colonization of Britain via two routes, although there are alternative explanations. For example, the 6 unique haplotypes in populations in England might occur elsewhere but were not detected in this study. Alternatively, the six unique haplotypes identified in England could have diverged from the shared haplotype in Scotland, Vosges, and Alps populations (haplotype 8; Figure 1b) since the LGM, although this seems highly unlikely given the short time period for one to three mutations to occur (Figure 1b). It is possible that these LGM populations were situated on land that is currently below sea level, at an edge of the glacier, or in sheltered low elevation microclimates on land. Multiple colonization events have also been shown in other taxa in the UK (Piertney et al., 2005), and the locations of cryptic refugia during the LGM are assumed to be ice‐free areas in southern England (Bocherens, Fogel, Tuross, & Zeder, 1995, Lister, 1984), northern Scotland (Bennett, 1984), and southern Ireland (Montgomery, Provan, McCabe, & Yalden, 2014). Evidence for cryptic refugia for insects in Britain also comes from cold‐adapted beetles (see Appendix S6; Buckland & Buckland, 2006), which currently have mountain or northern distributions in the UK, but were found as subfossil remains in southern England 18,000–26,000 years BP, providing evidence of cold‐adapted insects surviving in ice‐free locations in Britain in the LGM. It is, therefore, possible that the current population of *E. epiphron* in England survived elsewhere in Britain during the LGM as populations which no longer exist.

### 4.4 Future loss of unique genetic diversity in cold‐adapted species

High levels of genetic diversity are important in relation to the capacity for populations and species to adapt to changing environmental conditions, including climate change (Balint et al., 2011; Hoffmann & Sgro, 2011). Cold‐adapted species that have been shaped by diversification across mountain systems during the Pleistocene contain high levels of genetic diversity and unique populations, and are under threat from climate warming. Populations with unique genetic diversity may have evolved independently to be adapted to their local environment (Weeks, Stoklosa, & Hoffmann, 2016) and thus may be particularly vulnerable to future climatic changes. Our SDMs project loss of suitable climate for *E. epiphron* in many locations in Europe, especially in regions with predominantly low elevation populations and few opportunities to shift uphill to high elevation, which could result in loss of genetic diversity. However, our projections of range retraction do not take into account any potential of populations to adapt to warmer temperatures in situ (Franks & Hoffmann, 2012). Future loss of genetic diversity has also been predicted in other species (Alsos et al., 2012; Beatty & Provan, 2011; Yannic et al., 2014), and rates of loss of genetic diversity in wild populations since the industrial revolution (Leigh, Hendry, Vázquez‐Domínguez, & Friesen, 2019) are consistent with our projections.

### 4.5 Conservation interventions to mitigate climate‐driven genetic erosion

Conservation management and adaptation could protect cold‐adapted populations and safeguard unique genetic diversity from climate change (Mawdsley, O'Malley, & Ojima, 2009). Options include translocation or assisted colonization to areas that have, or are predicted to have, suitable climate and habitat in the future (Hoegh‐Guldberg et al., 2008). Translocations are a controversial topic due to the fear that translocated species may become “invasive” in their new ranges, posing threats to ecosystems including disturbance, disrupting ecological interactions, disease spread, competition, and extinctions (Ricciardi & Simberloff, 2009). However, others argue that the arrival of new species is typical of ecosystem changes in the Anthropocene and that translocations mirror colonizations occurring as a consequence of current environmental change (Thomas, 2011). Translocations of *E. epiphron* and other butterflies into unoccupied but climatically suitable areas have been successful (Cizek, Bakesova, Kuras, Benes, & Konvicka, 2003; Willis et al., 2009), and cold‐adapted insects may represent good targets for translocations given that the climate is rapidly deteriorating for them in many parts of their range, and they may find it difficult to colonize new areas across inhospitable landscapes (Thomas, 2011). For *E. epiphron*, our SDMs reveal areas in Scandinavia to be climatically suitable, although the species does not occur there, and climate is predicted to increase in suitability in future in Scandinavia for *E. epiphron* (Figure 3) and for other *Erebia* species (Settele et al., 2008). However, although Scandinavia may have suitable climate, it may not have the required habitat for *E. epiphron*. Local translocations within mountain systems that are currently occupied by *E. epiphron* could also be implemented, for example, moving individuals to areas of colder climate at higher elevation, or neighboring mountains which are too isolated for the species to colonize naturally. However, there may be very few areas of unoccupied but climatically suitable habitats within some mountain systems occupied by *E. epiphron*, particularly if the species already occurs at high elevations in these regions. Future work could include finer scale country‐specific SDMs with additional land use and genetic data on habitat availability could be used to locate areas for potential translocations.

As well as translocating individuals to new sites, it might be possible to consider translocating genes or “genetic rescue” by moving individuals among existing populations. Not only might this conserve unique genetic diversity at risk from local extinction of populations, but might increase the adaptive capacity of populations by increasing their genetic diversity (Aitken & Whitlock, 2013). This could involve moving warm‐adapted individuals into cooler populations to increase their adaptive capacity as the climate warms (Weeks et al., 2011). However, moving locally adapted populations may result in outbreeding depression and maladaptation, negatively impacting populations (Weeks et al., 2011), although some genetic rescue interventions have resulted in increases in populations, and alleles associated with local adaptation were not lost following gene flow (Fitzpatrick et al., 2020). Genetic conservation interventions for insects, and specifically butterflies, have been rarely implemented, although increasing habitat connectivity has led to genetic rescue of populations (Jangjoo, Matter, Roland, & Keyghobadi, 2016) and genetic data have been used to inform on reintroductions (Dinca et al., 2018). There is no evidence of attempted genetic rescue via translocations of butterflies, although translocating individuals is a genetic conservation strategy which may be important in ensuring future survival and adaptability of populations under climate change. As with translocations, these conservation options may also be controversial, but could remove the need for ongoing intervention and management at sites with declining populations (Weeks et al., 2011). We recommend that before the implementation of any climate adaptation strategy, populations are closely monitored to determine if populations are retracting and likely to become extinct in areas that are becoming too warm for the species. In addition, individual species’ assessments are required to assess the genetic diversity of populations and any local adaptation, which would determine the most appropriate conservation strategy.

## 5 CONCLUSIONS

The genetic diversification of cold‐adapted mountain species, as demonstrated in our study species *E. epiphron*, has been shaped by Pleistocene glaciations, the locations of long‐term survival of populations, and colonization patterns after the LGM, resulting in unique genetic diversity in isolated populations. Mountain and cold‐adapted species are vulnerable to future climate warming, and we predict *E. epiphron* will lose 38%–64% of its range in the future, especially at low elevations. The uniqueness of genetic diversity contained in these populations could be at risk depending on the severity of future climate change. Conservation strategies such as translocation could ensure the survival of these cold‐adapted species, but more research is needed on the likely effectiveness of such approaches.

## CONFLICT OF INTEREST

The authors declare no competing interests.

## AUTHOR CONTRIBUTION

**Melissa Minter:** Conceptualization (equal); Data curation (lead); Formal analysis (lead); Investigation (lead); Methodology (lead); Project administration (lead); Visualization (lead); Writing‐original draft (lead); Writing‐review & editing (lead). **Kanchon K. Dasmahapatra:** Conceptualization (lead); Funding acquisition (lead); Methodology (equal); Supervision (lead); Writing‐review & editing (equal). **Chris D. Thomas:** Conceptualization (lead); Funding acquisition (lead); Supervision (lead); Writing‐review & editing (equal). **Michael D. Morecroft:** Conceptualization (lead); Funding acquisition (lead); Supervision (lead); Writing‐review & editing (equal). **Athayde Tonhasca:** Conceptualization (lead); Funding acquisition (lead); Supervision (lead); Writing‐review & editing (equal). **Thomas Schmitt:** Conceptualization (equal); Data curation (equal); Methodology (supporting); Writing‐review & editing (equal). **Stefanos Siozios:** Data curation (equal); Methodology (supporting); Writing‐review & editing (supporting). **Jane K. Hill:** Conceptualization (lead); Funding acquisition (lead); Supervision (lead); Writing‐review & editing (equal).

## Supporting information

Appendix S1

Appendix S2‐S6

## Data Availability

GenBank and BOLD accession numbers for each mtDNA COI sequence can be found in Appendix S1.
