# Supplementary material for: Past, current, and potential future distributions of unique genetic diversity in a cold‐adapted mountain butterfly
Source: Ecol Evol. 2020 Sep 30;10(20):11155–68. doi: 10.1002/ece3.6755 (PMC7593187; doi:10.1002/ece3.6755)
Supplement: Supplementary file 2 — Appendix S2‐S6 [file ECE3-10-11155-s002.docx]

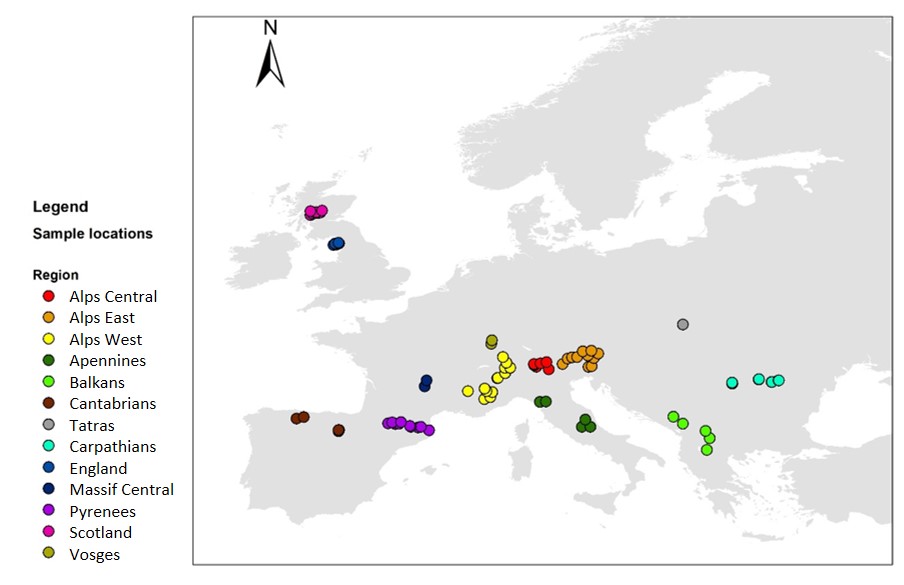


**Appendix S2:** Population locations for all individuals used in mtDNA analysis from 13 mountain regions.

**Appendix S3:** Bioclimatic variables used in SDMs to predict climate suitability for *E. epiphron*, extracted from mean temperature and precipitation data between 1970 and 2000 (<http://www.worldclim.org/>).’Cells’ are 2.5 arc minute (~4.5 km) resolution data extracted from within a 50km grid at the same spatial extent as distribution data. ‘Season’ refers to mean data from summer (June, July, August) and winter (December, January, February).

| Climate Variable | Location in 50km grid | Season |
| --- | --- | --- |
| Mean Temperature | Coldest cell | Winter (Coldest quarter) |
| Mean Temperature | Coldest cell | Summer (Warmest quarter) |
| Mean Temperature | Warmest cell | Winter (Coldest quarter) |
| Mean Temperature | Warmest cell | Summer (Warmest quarter) |
| Mean Precipitation | Wettest cell | Winter (Coldest quarter) |
| Mean Precipitation | Wettest cell | Summer (Warmest quarter) |
| Mean Precipitation | Driest cell | Winter (Coldest quarter) |
| Mean Precipitation | Driest cell | Summer (Warmest quarter) |

**Appendix S4:** All SDM outputs showing probability of climate suitability from present-day to 21,000 years ago (22 outputs in total). Probability values of occurrence for all panels are scaled from 0 (unsuitable, white) to 1 (suitable, black). Ice sheets (from (Hughes et al., 2016), blue shading) are present from 21,000 years BP to 10,000 years BP.

Present


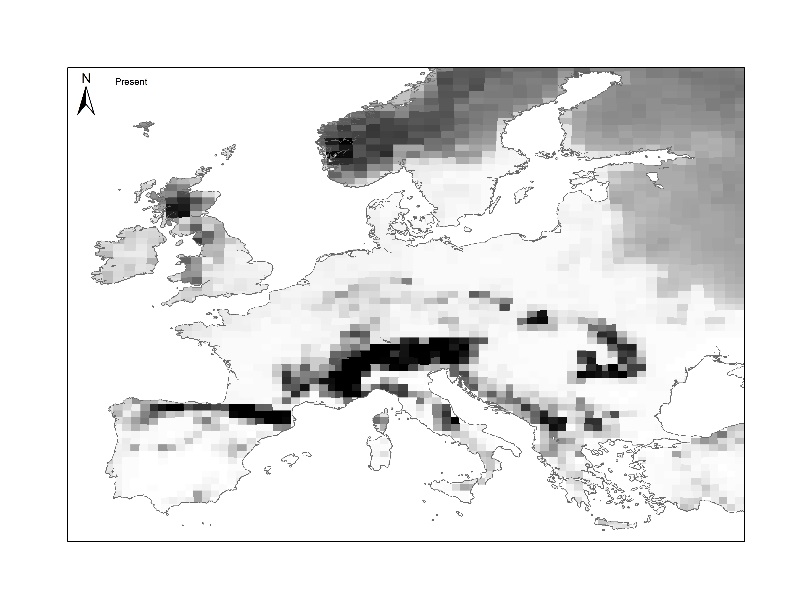


1,000 years BP


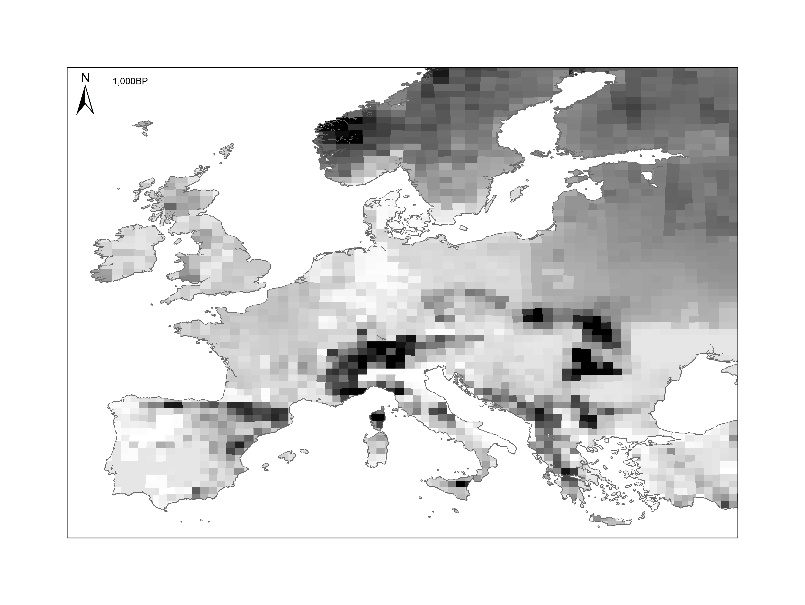


2,000 years BP


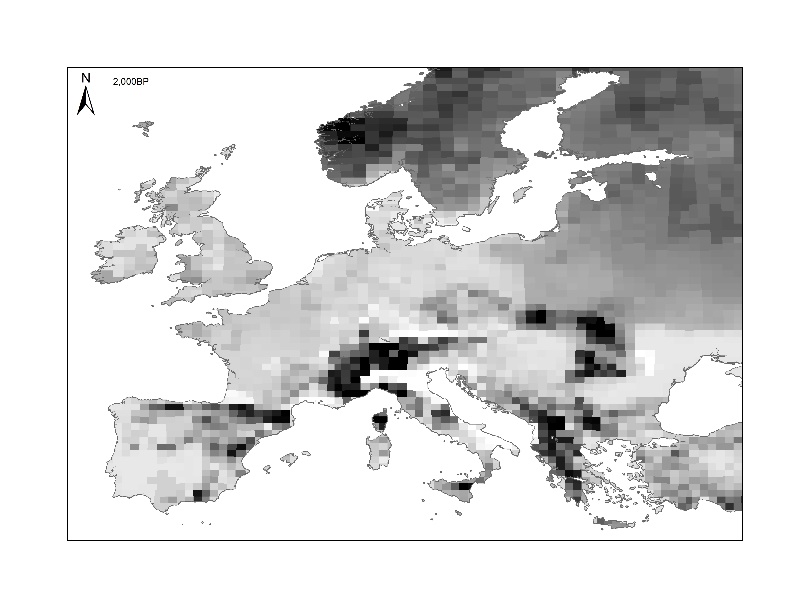


3,000 years BP


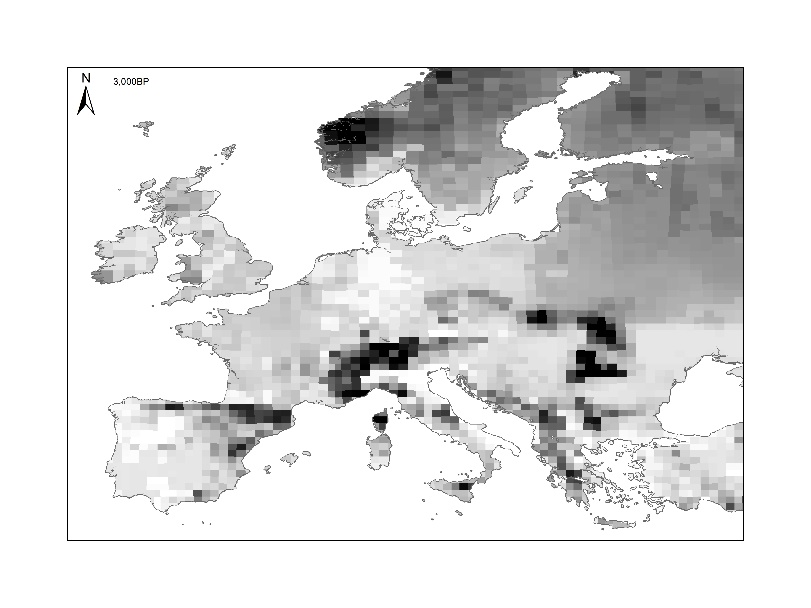


4,000 years BP


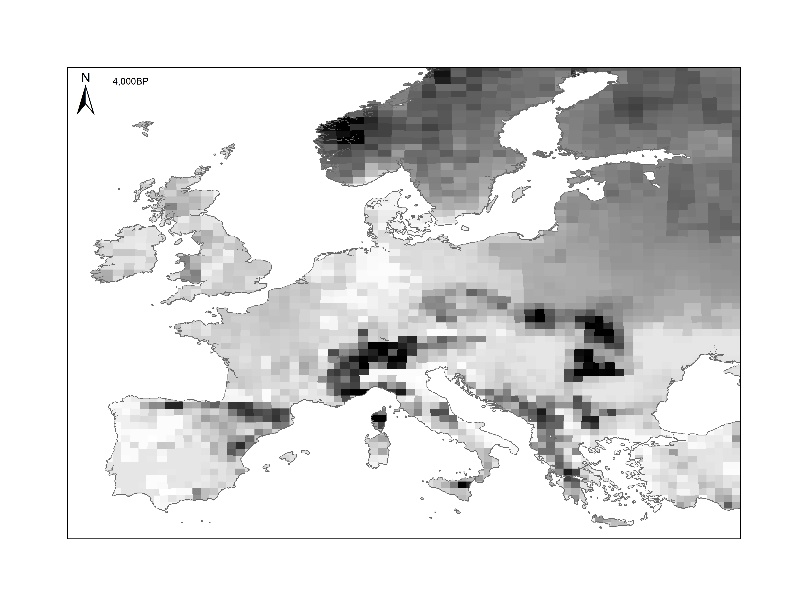


5,000 years BP


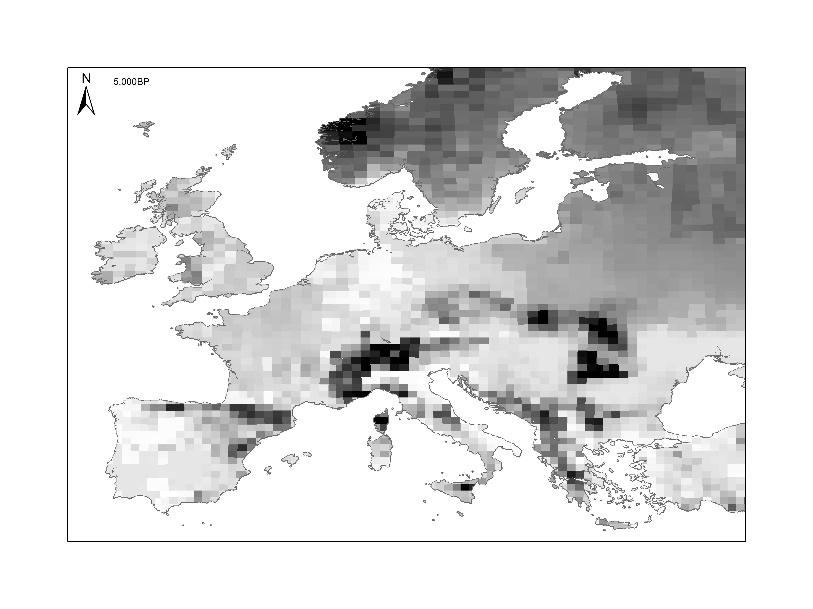


6,000 years BP


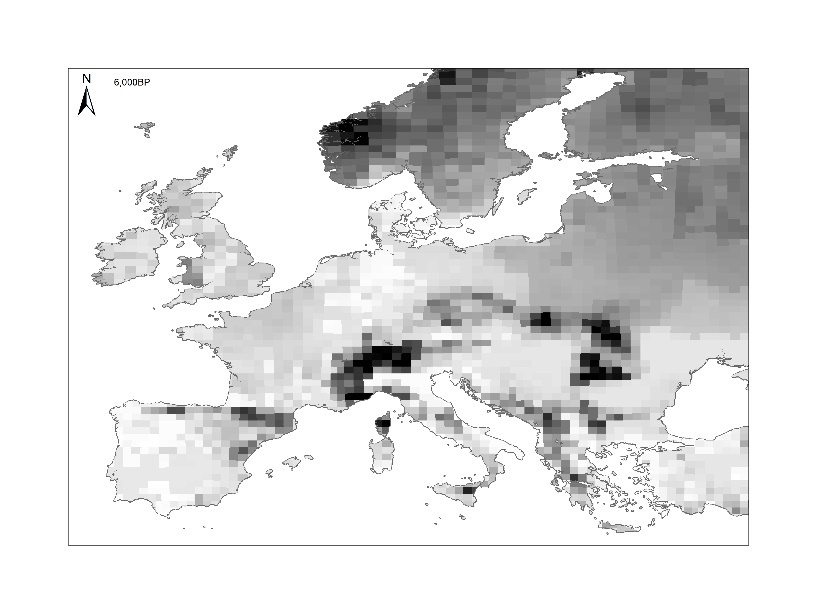


7,000 years BP


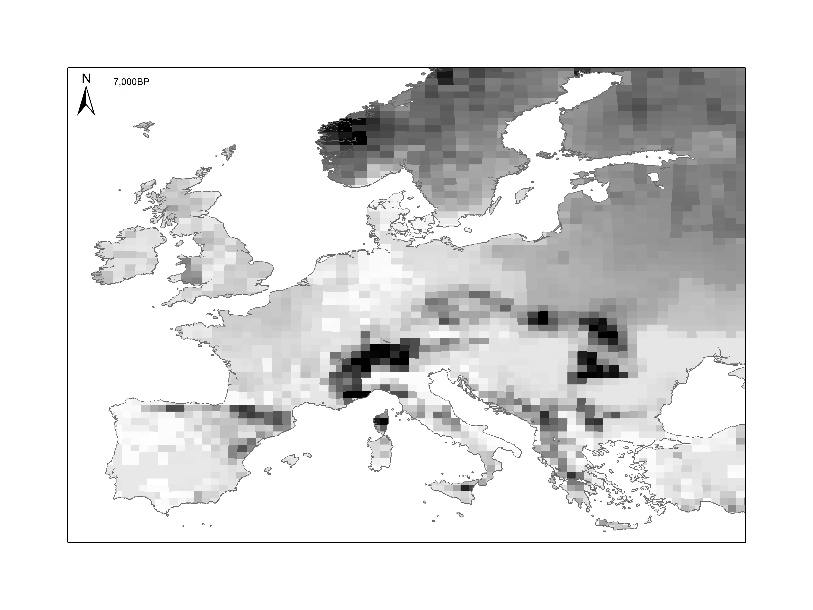


8,000 years BP


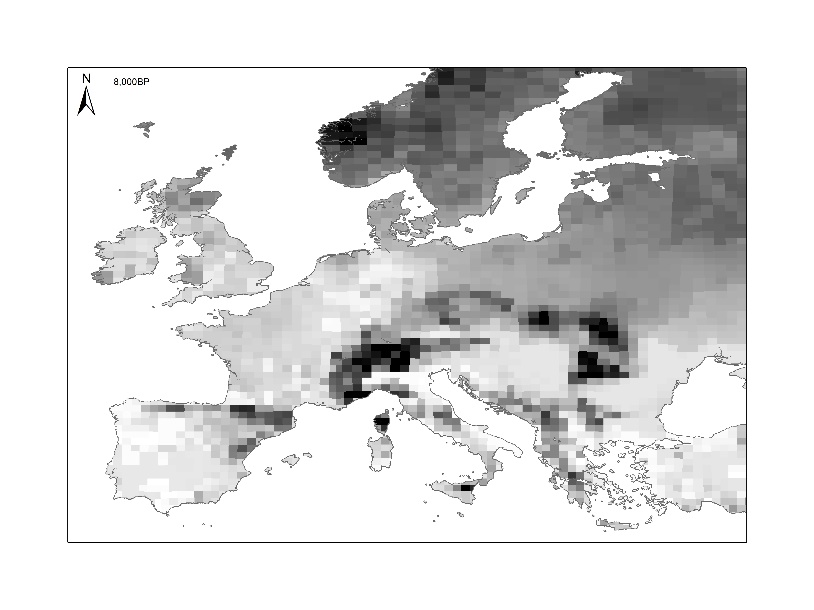


9,000 years BP


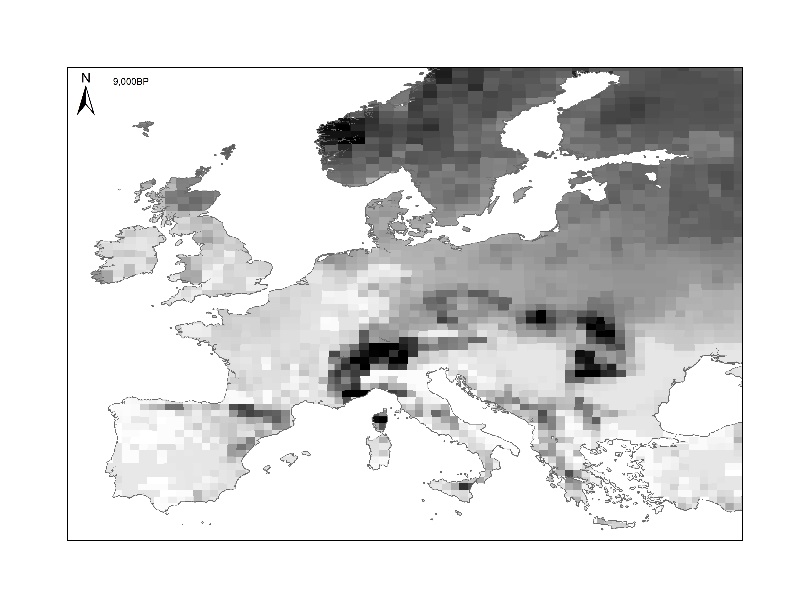


10,000 years BP


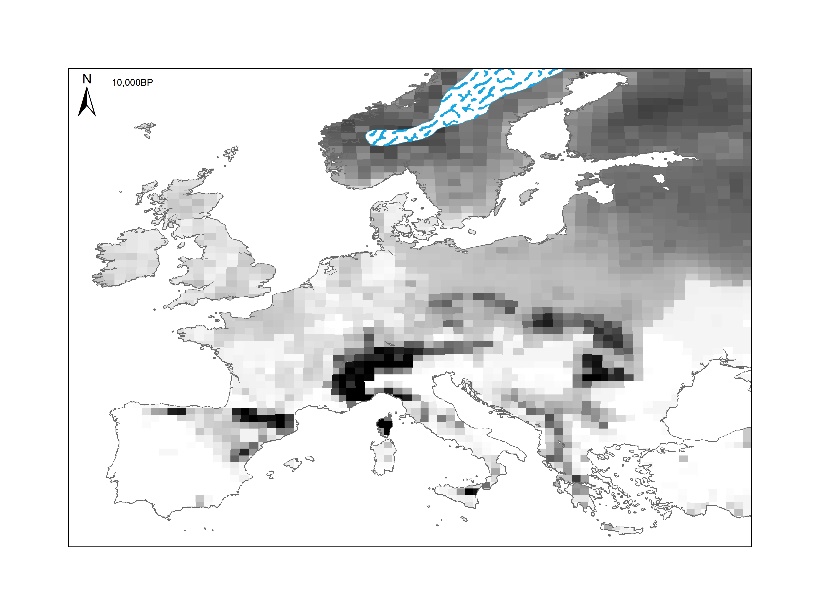


11,000 years BP


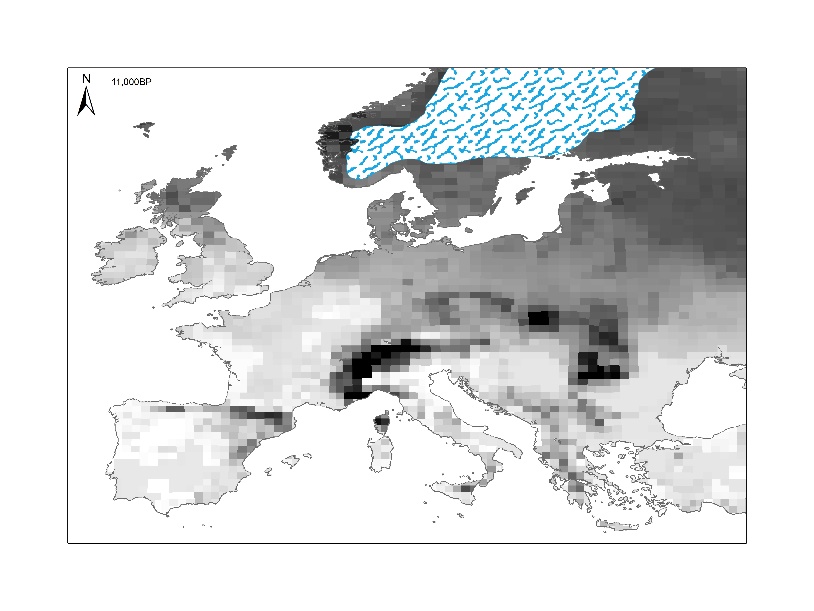


12,000 years BP


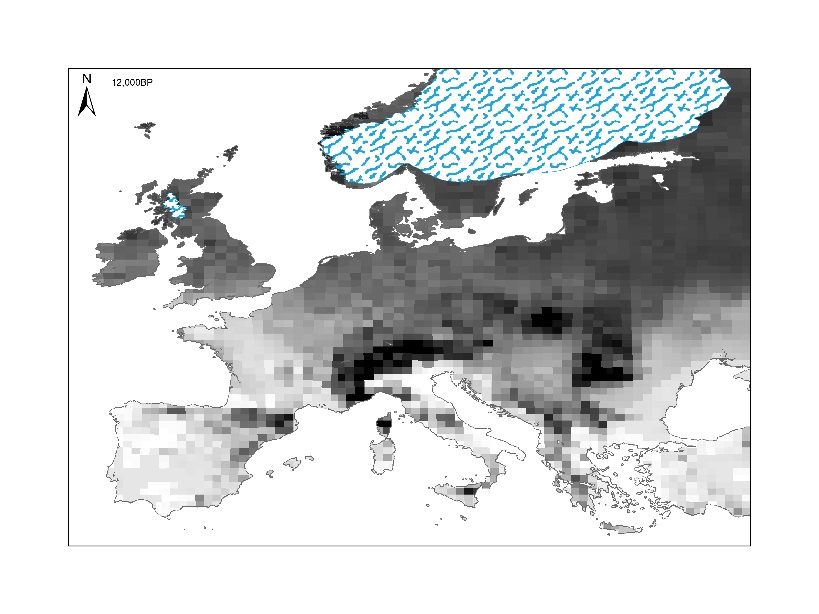


13,000 years BP


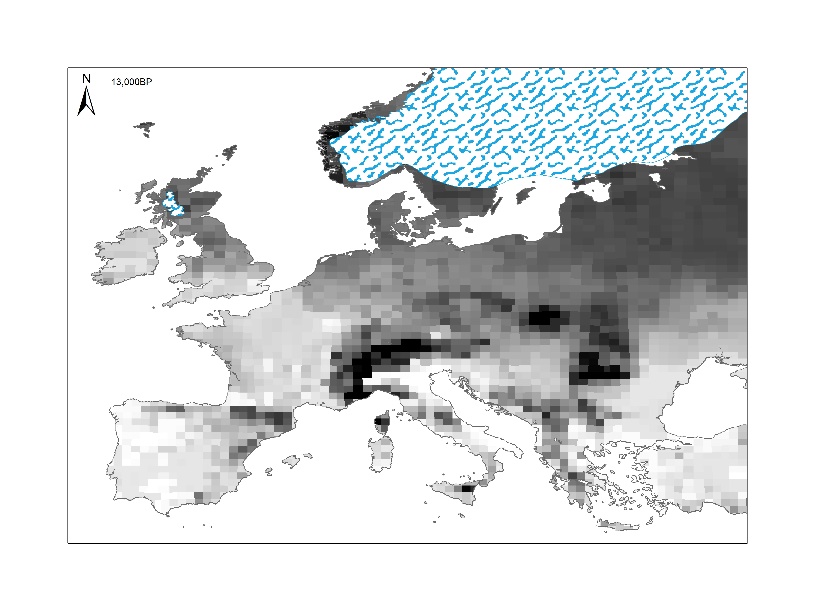


14,000 years BP


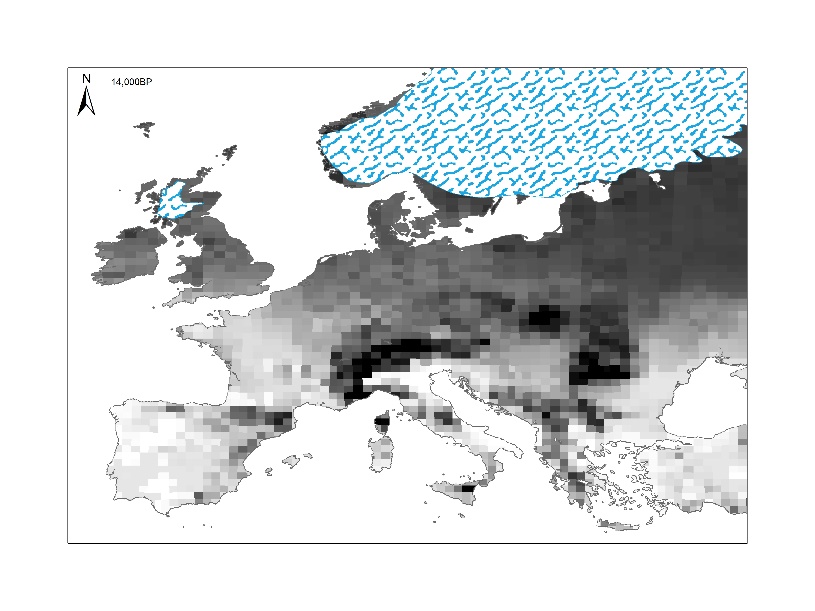


15,000 years BP


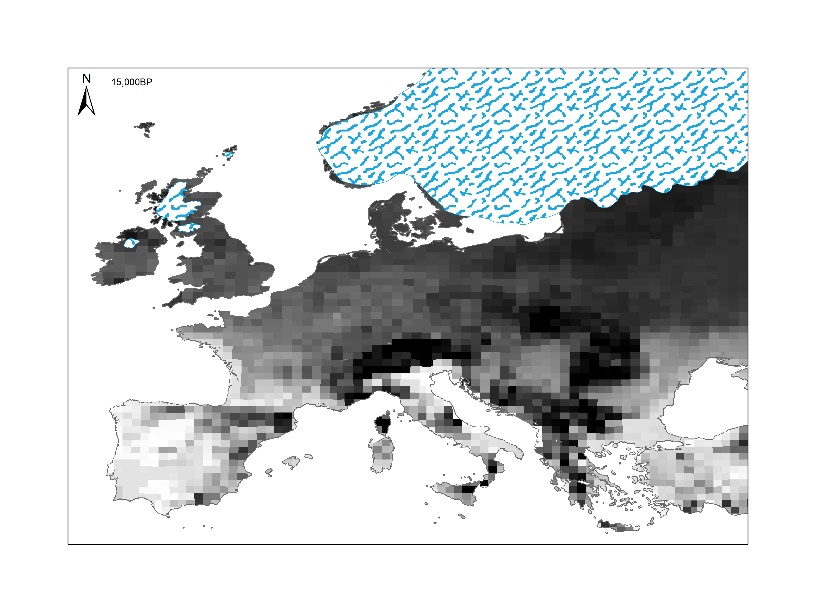


16,000 years BP


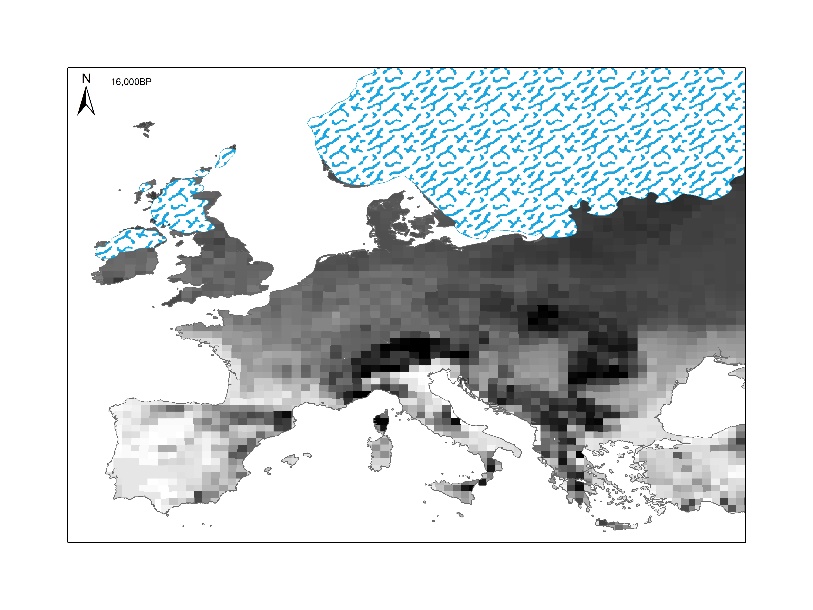


17,000 years BP


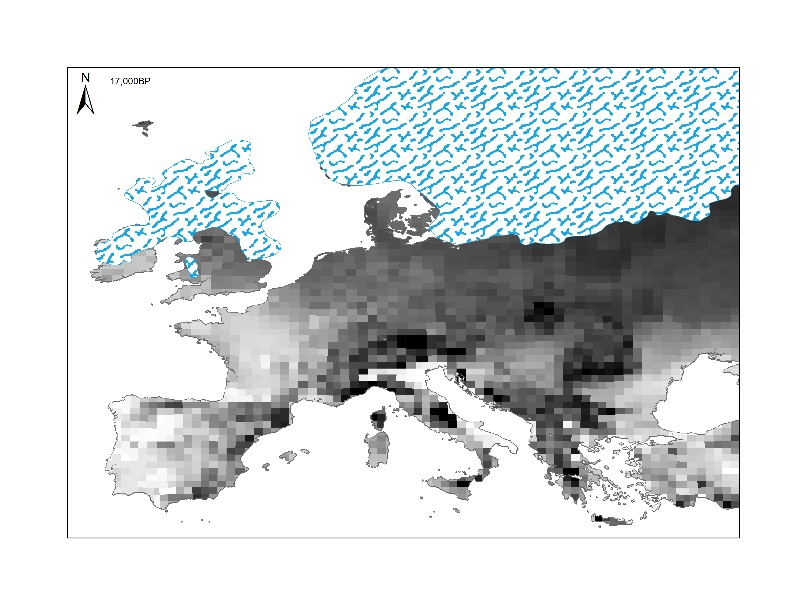


18,000 years BP


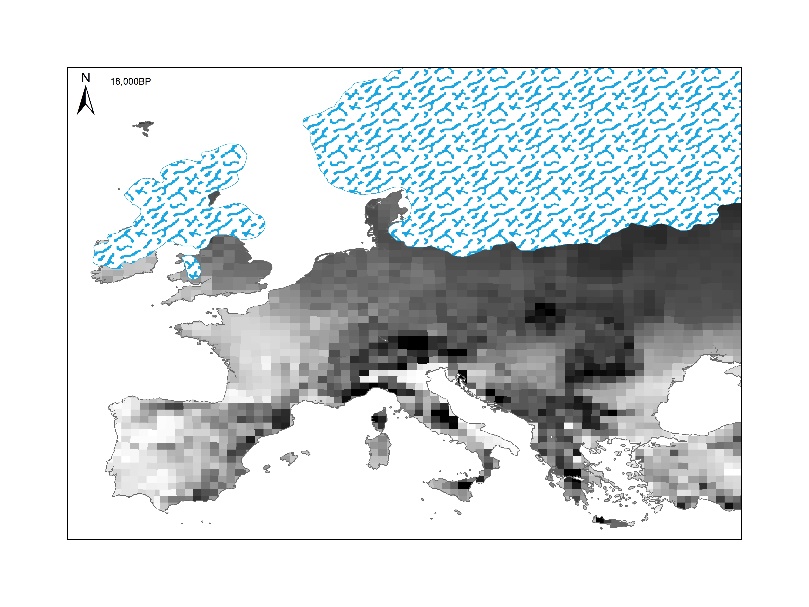


19,000 years BP


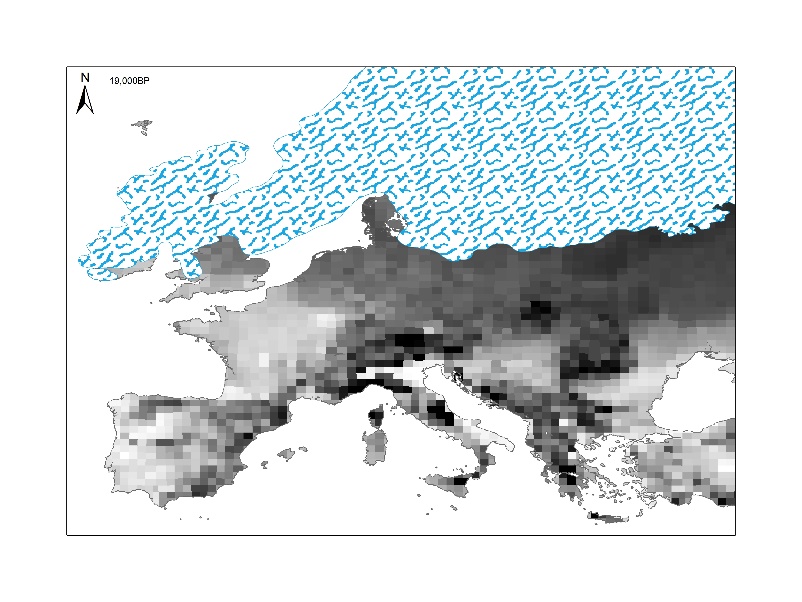


20,000 years BP


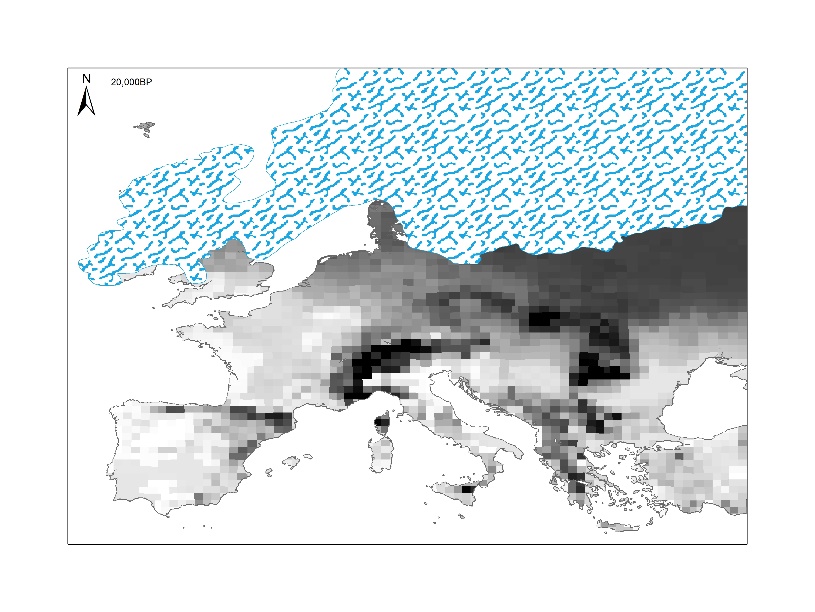


21,000 years BP


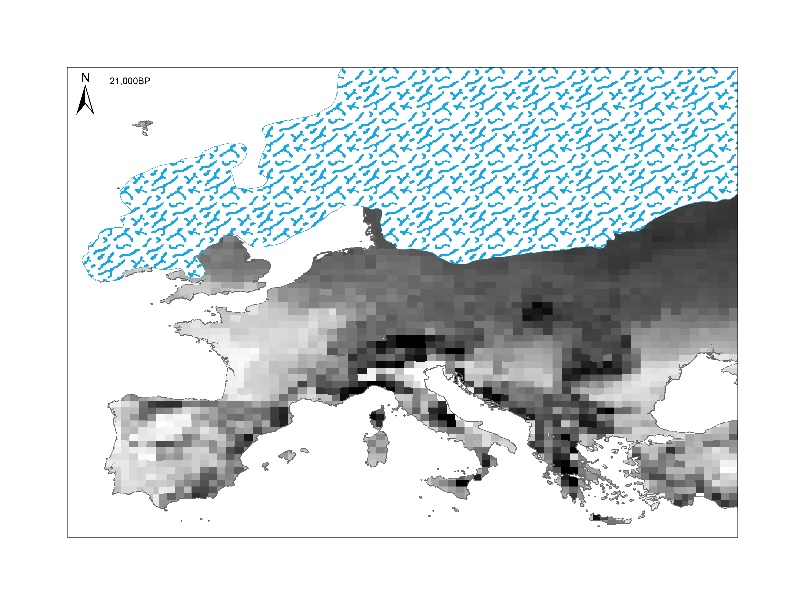


**Appendix S5**: CO1 phylogenetic tree of the *Erebia* genus, outgroups and *E. epiphron* populations. Phylogenetic tree analyses were performed in Beast using methods described by Pena, Witthauer, Kleckova, Fric, & Wahlberg, (2015). Outgroup and *Erebia* genus data were accessed from Genbank using accession numbers in Pena et al., (2015). Age of split between *Erebia* and sister taxa of 37.41 Myr (Pena et al., 2015) was used to calibrate the age split between *Erebia epiphron* and *E orientalis*. Scale bar represents age of tree in million years before present. Node number represent estimated age of node with blue error bars.

**
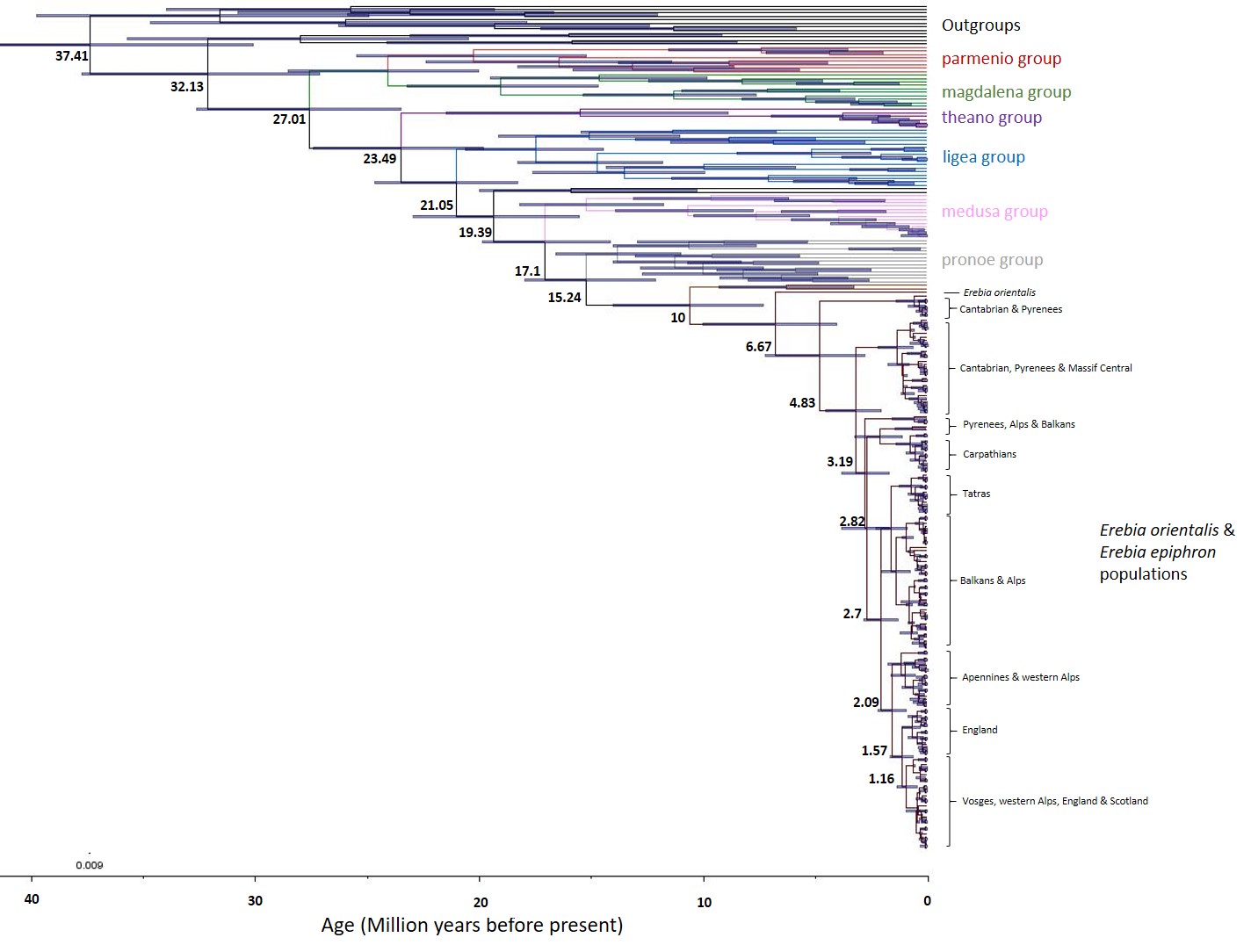
**

**Appendix S6**: Locations and ages of remains in the UK of the cool-adapted beetle species *Partobus septentrionis, Amara alpina, Amara quenseli* and *Notaris aethiops*, with corresponding most credible ice sheet extent. Beetle fossil data acquired from BugsCEP (Buckland & Buckland, 2006), ice sheet data from (Hughes, Gyllencreutz, Lohne, Mangerud, & Svendsen, 2016).


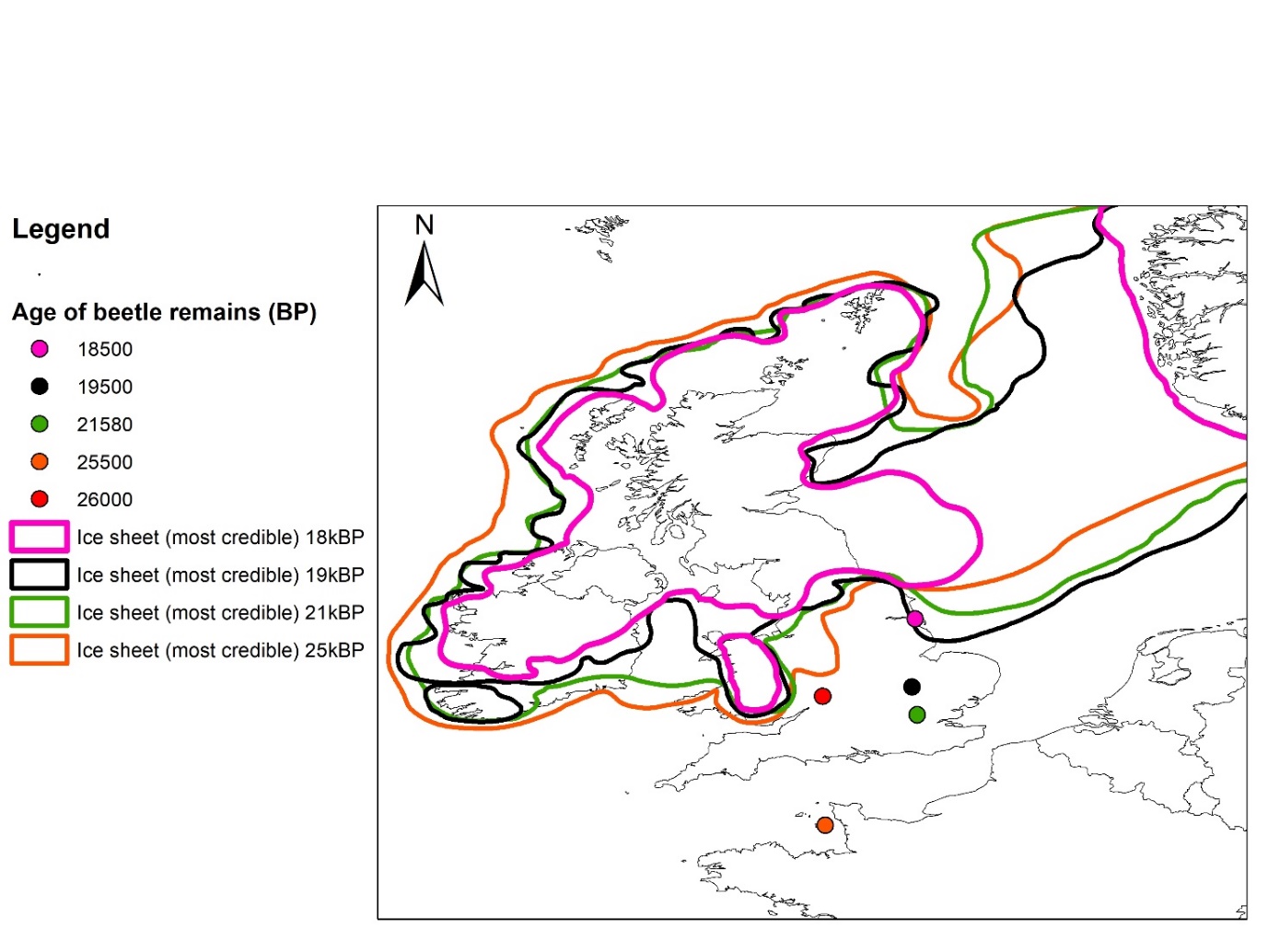


References

Buckland, P. I., & Buckland, P. C. (2006). BugsCEP Coleopteran Ecology Package. *IGBP PAGES/World Data Center for Paleoclimatology Data Contribution Series,* NOAA/NCDC Paleoclimatology Program, Boulder CO, USA. *http://www.bugscep.com*.

Hughes, A. L. C., Gyllencreutz, R., Lohne, O. S., Mangerud, J., & Svendsen, J. I. (2016). The last Eurasian ice sheets - a chronological database and time-slice reconstruction, DATED-1. *Boreas,* 45(1). doi:10.1111/bor.12142

Pena, C., Witthauer, H., Kleckova, I., Fric, Z., & Wahlberg, N. (2015). Adaptive radiations in butterflies: evolutionary history of the genus Erebia (Nymphalidae: Satyrinae). *Biological Journal of the Linnean Society,* 116(2), 449-467. doi:10.1111/bij.12597
